# Indirect influence of soil enzymes and their stoichiometry on soil organic carbon response to warming and nitrogen deposition in the Tibetan Plateau alpine meadow

**DOI:** 10.3389/fmicb.2024.1381891

**Published:** 2024-04-17

**Authors:** Xiang Xuemei, De Kejia, Lin Weishan, Feng Tingxu, Li Fei, Wei Xijie

**Affiliations:** College of Animal Husbandry and Veterinary Science, Qinghai University, Xining, China

**Keywords:** warming, nitrogen deposition, soil organic carbon, enzyme activity, microbial community, Tibetan Plateau, alpine meadows

## Abstract

Despite extensive research on the impact of warming and nitrogen deposition on soil organic carbon components, the response mechanisms of microbial community composition and enzyme activity to soil organic carbon remain poorly understood. This study investigated the effects of warming and nitrogen deposition on soil organic carbon components in the Tibetan Plateau alpine meadow and elucidated the regulatory mechanisms of microbial characteristics, including soil microbial community, enzyme activity, and stoichiometry, on organic carbon components. Results indicated that both warming and nitrogen deposition significantly increased soil organic carbon, readily oxidizable carbon, dissolved organic carbon, and microbial biomass carbon. The interaction between warming and nitrogen deposition influenced soil carbon components, with soil organic carbon, readily oxidizable carbon, and dissolved organic carbon reaching maximum values in the W0N32 treatment, while microbial biomass carbon peaked in the W3N32 treatment. Warming and nitrogen deposition also significantly increased soil Cellobiohydrolase, β-1,4-N-acetylglucosaminidase, leucine aminopeptidase, and alkaline phosphatase. Warming decreased the soil enzyme C: N ratio and C:P ratio but increased the soil enzyme N:P ratio, while nitrogen deposition had the opposite effect. The bacterial Chao1 index and Shannon index increased significantly under warming conditions, particularly in the N32 treatment, whereas there were no significant changes in the fungal Chao1 index and Shannon index with warming and nitrogen addition. Structural equation modeling revealed that soil organic carbon components were directly influenced by the negative impact of warming and the positive impact of nitrogen deposition. Furthermore, warming and nitrogen deposition altered soil bacterial community composition, specifically *Gemmatimonadota* and *Nitrospirota*, resulting in a positive impact on soil enzyme activity, particularly soil alkaline phosphatase and β-xylosidase, and enzyme stoichiometry, including N:P and C:P ratios. In summary, changes in soil organic carbon components under warming and nitrogen deposition in the alpine meadows of the Tibetan Plateau primarily depend on the composition of soil bacterial communities, soil enzyme activity, and stoichiometric characteristics.

## Introduction

The alpine grasslands of the Qinghai-Tibet Plateau encompass approximately 40% of China’s grassland area. This region holds global ecological significance, serving as a repository for unique biodiversity and genetic resources crucial for high-altitude organisms. Additionally, it plays a vital role in providing ecosystem services such as biodiversity maintenance, regulation of nutrient cycling, hydrological function, and carbon storage (Fu et al., 2021). Among the grassland types in this area, alpine meadows are prominent, especially in high-altitude regions, which are particularly susceptible to the effects of climate change (Liu S. B. et al., 2018). Projections indicate that temperatures on the Qinghai-Tibet Plateau are expected to rise by 1.5–2.9°C between 2030 and 2099, with nitrogen deposition averaging 8.7–13.8 kg N ha^−1^ yr.^−1^ (Liu X. D. et al., 2009; Pang et al., 2019). Climate warming and nitrogen deposition are intertwined components of global climate change (Sun et al., 2021). Climate warming can escalate the emission of reactive nitrogen into the atmosphere, resulting in lower nitrogen use efficiency and atmospheric pollution (Li et al., 2024). However, the increase in atmospheric reactive nitrogen can directly or indirectly contribute to further climate warming (Ma et al., 2022). Extensive experimentation has demonstrated that climate warming and nitrogen deposition significantly impact the structure and function of grassland ecosystems, leading to declines in biodiversity, alterations in carbon cycling, and changes in grassland multifunctionality (Su et al., 2022).

Soil carbon storage represents the largest carbon reservoir in terrestrial ecosystems, surpassing vegetation and atmospheric carbon reservoirs by 2–3 times (Delgado-Baquerizo et al., 2017). Soil organic carbon constitutes the primary component of soil carbon storage and can be categorized into the biologically active, unstable organic carbon pool, and the long-term stable organic carbon pool based on its decomposition degree and turnover rate (Li et al., 2018). Unstable organic carbon, including labile organic carbon, soluble organic carbon, microbial carbon, and other carbon fractions, plays a crucial role in regulating soil nutrient flow and carbon-nitrogen fluxes (Cong et al., 2020). Minor changes in grassland soil carbon storage, such as increased soil respiration, can significantly impact atmospheric carbon dioxide production, thereby exacerbating global climate warming through positive feedback mechanisms (Nottingham et al., 2020). Therefore, understanding the response of soil organic carbon fractions to warming is crucial for exploring the carbon cycle in grassland ecosystems. Evidence suggests that experimental warming can have negative effects (Wang et al., 2019), positive effects (Zheng et al., 2020), or no effect (Tian et al., 2021) on soil organic carbon fractions. Due to the fundamental coupling of carbon and nitrogen cycles in terrestrial ecosystems, enhanced nitrogen availability can alter ecosystem carbon cycling and accumulation processes (Yang et al., 2011). Previous studies have indicated that nitrogen deposition may increase (Wang et al., 2022), decrease (Zhong et al., 2017), or have no effect on organic carbon content (Balesdent et al., 2000). Variations in grassland vegetation types, nitrogen addition rates, warming magnitudes, and experimental durations can influence soil organic carbon fractions (Lu et al., 2011; Luo et al., 2019). While most previous research on organic carbon fractions has focused solely on individual climatic factors, few studies have investigated the effects of multiple climate factors on organic carbon fractions. Therefore, exploring differences in soil organic carbon fractions in response to nitrogen deposition and warming is a crucial direction for increasing soil carbon storage and reducing greenhouse gas emissions under future climate change conditions.

Soil microbes represent indispensable components of terrestrial ecosystems, fulfilling crucial roles in promoting soil organic matter turnover and enhancing soil nutrient mineralization rates in the soil carbon cycle (Van Der Heijden et al., 2008). Research indicates that under elevated temperatures and nitrogen deposition, soil microbes not only release carbon into the air through heterotrophic decomposition but also metabolize exogenous carbon into certain substances and sequester them in the soil through synthetic processes (Tamura and Tharayil, 2014). Additionally, soil microbes can influence soil aggregate stability by secreting carbon-transforming enzymes (Trivedi et al., 2016). Consequently, any alterations in the diversity, composition, and potential functions of microbial communities may affect the direction of carbon mineralization (Ibrahim et al., 2021). Soil microbial extracellular enzymes serve as potential indicators of microbial function, playing critical roles in the degradation, transformation, and mineralization of soil organic matter in the carbon cycle (Nannipieri et al., 2018). Studies indicate that soil enzymes produced by soil microbes, such as β-glucosidase and β-xylosidase activities, regulate carbon transformation processes by degrading different molecules or depolymerizing large molecular substrates (Duan et al., 2021). Furthermore, nitrogen enrichment and climate warming may disrupt soil element balances, affecting the limited resources available for soil microbial metabolism (Steinweg et al., 2013). Microbial enzyme stoichiometry is recognized as an effective tool for assessing the environmental drivers of microbial metabolism. Research has demonstrated that changes in soil organic carbon induced by warming are associated with microbial enzyme stoichiometry (Stark et al., 2018). Therefore, microbial enzyme stoichiometry plays a pivotal role in controlling soil carbon cycling (Buchkowski et al., 2015). While there is abundant research on the response of soil organic carbon fractions to warming and nitrogen deposition (Ding et al., 2019; Chen et al., 2020), most studies overlook the regulatory mechanisms of soil microbial characteristics on soil carbon fractions.

Investigating the changes in soil organic carbon fractions and their regulatory mechanisms under nitrogen deposition and climate warming in the alpine meadows of the Qinghai-Tibet Plateau is crucial for enhancing our understanding of organic carbon cycling mechanisms in these ecosystems under future climate change. We hypothesize the following: (1) Both warming and nitrogen deposition will significantly affect soil organic carbon fractions, enzyme activity, stoichiometry, microbial diversity, and composition. However, soil enzyme stoichiometry will respond differently to warming and nitrogen deposition. (2) Soil organic carbon fractions will experience distinct impacts from warming and nitrogen deposition, with soil bacterial communities exerting a greater influence than fungi. Additionally, soil enzyme activity and stoichiometry are pivotal in regulating organic carbon composition.

## Materials and methods

### Study area overview

The study was conducted at the Chengdu Zi Station of the Three-River Source Grassland Ecosystem National Field Scientific Observation and Research Station in Qinghai Province (33° 24′30″N, 97° 18′00″E), situated at an elevation of 4,270 m. The area exhibits a typical plateau continental climate, characterized by an annual average temperature ranging from −5.6°C to 3.8°C and an average annual precipitation of 562.2 mm. The majority of the precipitation, approximately 75% of the annual total, falls during the peak growing season for grasses from July to September. Dominant vegetation species include *Kobresia humilis* (C.A.Mey ex Trauvt) Serg., *Kobresia pygmaea* Clarke, *Elymus nutans* Griseb., and *Poa annua* L.

### Experimental design

**Figure 1:**
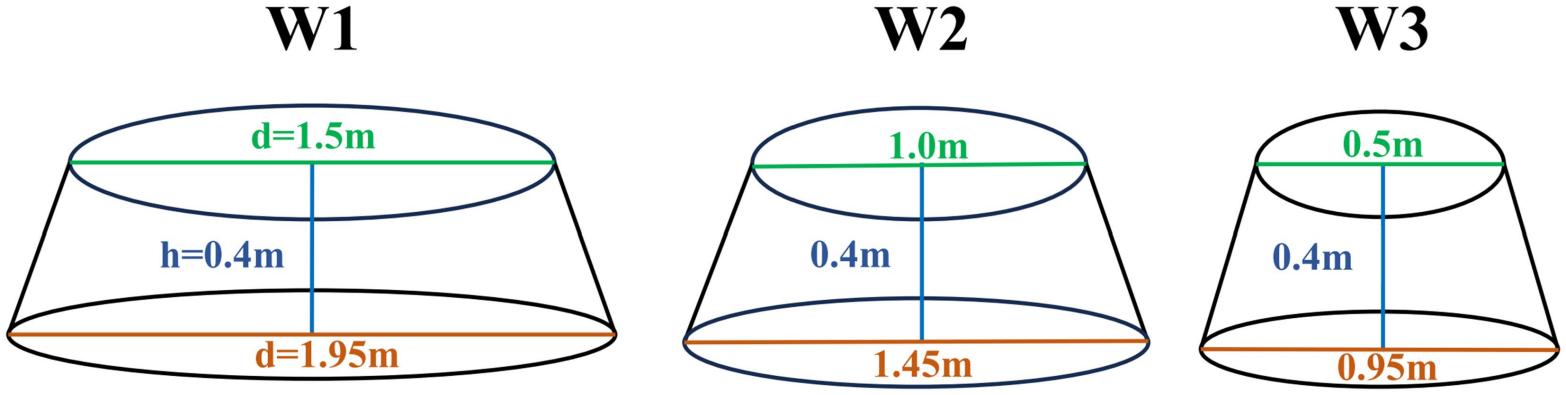
Schematic diagram of warming facility specifications.

The field experiment for this study was conducted in May 2023 within a fenced flat area measuring 50 × 50 m^2^. Projections suggest that the Qinghai-Tibet Plateau will face additional warming of up to 2.0°C by 2035 and up to 4.9°C by 2100 (Peng et al., 2017). Consequently, the study comprised four temperature treatments (non-warming and three warming treatments) established within plots. Three temperature gradients were created using Open-Top Chambers (OTCs) to mitigate the volume effect (Table 1; Figure 1). Soil and air temperatures were continuously monitored using HOBOS-TMB-M006 Temperature Smart Sensors (HOBO, United States).

**Table 1:** Specifications and performance of warming facility.

Warming gradient (°C)	Top diameter (m)	Bottom diameter (m)	Height (m)	Temperature (°C)	
				10 cm above ground air temperature	5 cm below ground soil temperature
W1	1.5	1.95	0.4	0.47	0.61
W2	1	1.45	0.4	0.92	1.09
W3	0.5	0.95	0.4	1.44	1.95

W1, W2, and W3 denote the warming conditions relative to W0.

Environmental nitrogen deposition on the Qinghai-Tibet Plateau is estimated to be approximately 8 kg N ha^−1^ yr.^−1^, predominantly in the form of NH4-N and NO3-N (Zhan et al., 2019). Hence, three nitrogen deposition levels were chosen to simulate future atmospheric N deposition: N0 (0 kg ha^−1^ yr.^−1^), N16 (16 kg N ha^−1^ yr.^−1^), and N32 (32 kg N ha^−1^ yr.^−1^). Ammonium nitrate was utilized to simulate nitrogen deposition during the growing season, dissolved in water, and uniformly sprayed onto the plots, with an equivalent amount of water sprayed in the control. The experiment followed a randomized complete block design, with four replicates in each plot and 12 treatment levels per subplot, including W0N0 (no warming or nitrogen addition, CK), W0N8, W0N32, W1N0, W1N8, W1N32, W2N0, W2N8, W2N32, W3N0, W3N8, and W3N32, resulting in a total of 48 plots.

### Sample collection

Sampling was conducted during the plant growing season in August 2023, with samples randomly taken from each treatment within 0.5 × 0.5 m quadrats. From each quadrat, five soil cores with a diameter of 3 cm and a depth of 30 cm were randomly collected. These cores were combined to create one soil sample. Plant roots and stones were eliminated using a 2 mm mesh sieve. The sample was then divided into two subsamples: one stored at −80°C for subsequent microbial analysis, and the other air-dried naturally for determining physicochemical properties.

### Soil sample analysis

#### Soil organic carbon components

Soil organic carbon (SOC) was quantified employing the potassium dichromate oxidation method with external heating. Microbial biomass carbon (MBC) was extracted using the chloroform fumigation method and analyzed using a carbon and nitrogen analyzer. Dissolved organic carbon (DOC) was determined via a TOC analyzer following deionized water extraction. The content of readily oxidizable carbon (ROC) in soil was measured utilizing the potassium permanganate oxidation method with UV spectrophotometry.

#### Soil enzyme activity and stoichiometry

Enzymes involved in carbon cycling [cellobiohydrolase (CBH), β-xylosidase (βX)], nitrogen cycling [β-1,4-N-acetylglucosaminidase (NAG), leucine aminopeptidase (LAP)], and phosphorus cycling [alkaline phosphatase (AKP/ALP)] were measured using a 96-well microplate fluorescence assay (Peng and Wang, 2016). After incubating soil with substrates for 4 h, 10 μL of NaOH was added to adjust the pH of the reaction mixture for optimal fluorescence values. Fluorescence values were then detected using a multifunctional enzyme analyzer (Tecan Infinite M200, Austria), with excitation and emission wavelengths of 365 nm and 450 nm, respectively. Enzyme activity was expressed as μmol·g^−1^·h^−1^. Soil extracellular enzyme C: N, C:P, and N:P stoichiometric ratios were calculated as ln (CBH + βX): ln (NAG + LAP), ln (CBH + βX): ln (AKP/ALP), and ln (NAG + LAP): ln (AKP/ALP), respectively (Xu et al., 2020).

### Soil microbial community composition

Approximately 0.5 g of refrigerated soil samples were utilized for total DNA extraction following the manufacturer’s protocol of the DNA extraction kit (Omega Bio-tek, Norcross, GA, United States). The concentration and purity of DNA were assessed using the NanoDrop 2000 spectrophotometer (Thermo Scientific Inc., Waltham, MA, United States). PCR amplification targeting the V3-V4 region of bacterial 16S rRNA (with primers 341F: 5′-CCTACGGGNGGCWGCAG-3′ and 805R: 5′-GACTACHVGGGTATCTAATCC-3′) and the ITS2 region of fungal ITS (with primers ITS-1F: 5′-GTGARTCATCRARTYTTTG-3′ and ITS-2: 5′-TCCTSCGCTTATTGATATGC-3′) was conducted using the ABI GeneAmp^®^9700 PCR thermal cycler (ABI, CA, United States). The PCR reaction system (20 μL) included 4 μL of 5 × TransStartFastPfu buffer, 0.8 μL of each primer, 2 μL of 2.5 mM dNTPs, 0.4 μL of TransStart FastPfu DNA polymerase, 10 ng of template DNA, and sterilized ddH2O to make up the volume. The PCR program comprised an initial denaturation at 95°C for 3 min, followed by 32 cycles of denaturation at 95°C for 30 s, annealing at 56°C for 30 s, extension at 72°C for 45 s, and a final extension at 72°C for 10 min. Each sample was run in triplicate. PCR products were purified from 2% agarose gels using the AxyPrep DNA Gel Extraction Kit (Axygen Biosciences, Union City, CA, United States) following the manufacturer’s instructions and quantified using the Quantus^TM^ Fluorometer (Promega, United States). The Illumina NovaSeq 6000 platform was employed for paired-end sequencing (PE250) of 16S and ITS amplicons to generate raw data. To assess sample diversity, clean data were imported into QIIME2 for DADA2-based filtering, quality control, chimera removal, merging of paired-end reads, and denoising to generate amplicon sequence variants (ASVs). For taxonomic classification, the bacterial classifier was trained using the Silva138 99% clustered sequences of the V3-V4 region, while the fungal classifier was trained using the UNITE database (version 8.0). The trained classifiers were then used to assign taxonomic information to the ASVs, resulting in the microbial community composition of each sample.

### Statistical analysis

To assess the impacts of warming, nitrogen deposition, and their interaction on soil organic carbon fractions, enzyme activities, and the diversity and richness of soil bacterial and fungal communities, a linear mixed-effects model was employed, with warming and nitrogen deposition as fixed factors and site as a random factor. Furthermore, one-way analysis of variance (ANOVA) with Tukey’s test was utilized to compare differences between nitrogen and warming treatments, with a significance level set at *p* < 0.05. All results are expressed as mean ± standard deviation. Chao1 and Shannon diversity indices were calculated using Qiime software (Version 1.9.0). Soil microbial beta diversity was assessed using the Anosim test to determine if between-group differences were greater than within-group differences, indicating the significance of grouping. *R*-values, ranging from −1 to 1, were obtained, with R > 0 indicating greater between-group differences and R < 0 indicating the opposite. Larger |R| values denote greater differences, with *p*-values indicating the confidence level of statistical analysis (*p* < 0.05 indicates significance). Canoco 5 was utilized to explore the principal factors affecting soil organic carbon under nitrogen deposition and warming conditions, incorporating soil microbial diversity and composition. A structural equation model (SEM) in SPSS Amos 22 software was employed to establish links among soil enzyme activities, microbial diversity, dominant bacterial phyla, and soil organic carbon fractions under warming and nitrogen deposition conditions. Variables for SEM were selected based on Monte Carlo permutation tests conducted in redundancy analysis, with model fit assessed using CMIN/Df, *p*-values, goodness-of-fit index (GFI), and root mean square error of approximation (RMSEA) to determine the optimal-fit model.

## Results

### Soil organic carbon fractions

**Figure 2:**
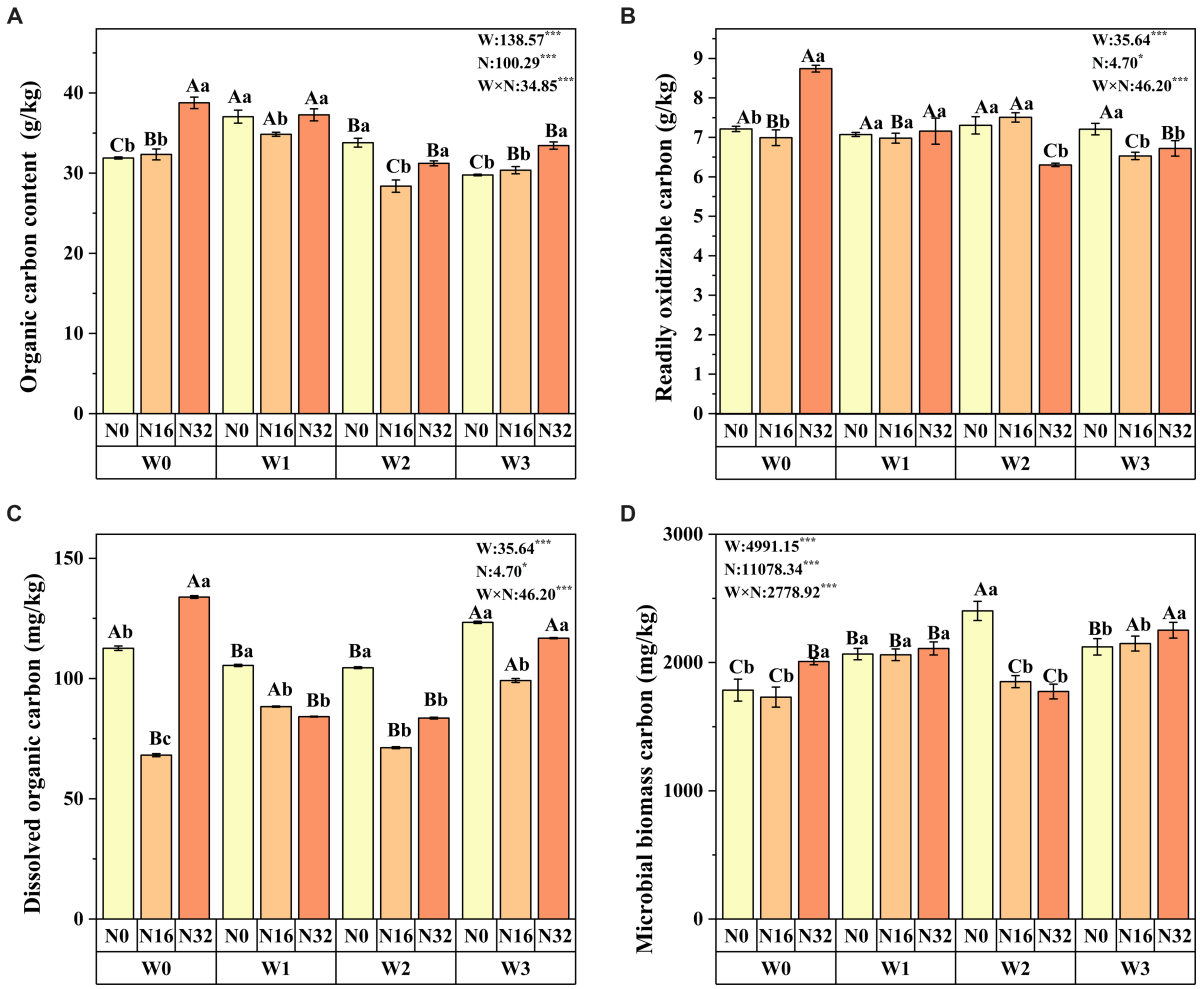
Impact of warming, nitrogen deposition, and their interaction on soil carbon fractions. (A–D) respectively soil organic carbon, readily oxidizable organic carbon content, dissolved organic carbon and microbial biomass carbon. Lowercase letters indicate significant differences among nitrogenlevels within the same warming treatment, whereas uppercase letters denote significant differences among warming treatment nitrogen level (*n* = 4). W represents the effect of the warming treatment, N represents the effect of the nitrogen treatment, and W*N represents the interaction effect of warming and nitrogen treatment. *p < 0.05; **p < 0.01; ***p < 0.001; ns indicates not significant.

Warming significantly affects soil organic carbon, readily oxidizable organic carbon content, dissolved organic carbon, and microbial biomass carbon. Similarly, nitrogen deposition also significantly impacts these soil carbon fractions. Moreover, the interaction between warming and nitrogen deposition influences all soil carbon fractions (Figure 2). Compared to the control group (W0), soil organic carbon increased significantly by 1.40 and 9.30% in the W1 and W3 treatments, respectively. Readily oxidizable organic carbon content increased by 1.27% in the W2 treatment, while dissolved organic carbon increased by 9.56% in the W3N0 treatment. Microbial carbon increased by 1.27 and 15.75% in the W2 and W3 treatments, respectively. Similarly, compared to the control group (N0), soil organic carbon increased significantly by 1.40 and 21.59% in the N16 and N32 treatments, respectively. Readily oxidizable organic carbon content increased by 21.18% in the N32 treatment, and dissolved organic carbon increased by 18.92% in the N36 treatment. Microbial carbon increased by 12.48% in the N32 treatment (Figure 2). Notably, soil organic carbon, readily oxidizable organic carbon content, dissolved organic carbon, and microbial carbon reached their maximum values in the W0N32 treatment, whereas microbial carbon peaked in the W3N32 treatment. The proportions of dissolved organic carbon, microbial biomass carbon, and readily oxidizable carbon in total soil organic carbon were significantly influenced by warming, nitrogen deposition, and their interaction. Particularly, readily oxidizable carbon constituted the main part (26.5–18.0%) of soil organic carbon, while dissolved organic carbon comprised the least (0.41–0.21%) (Supplementary Table S1).

### Soil enzyme activity and stoichiometry

**Figure 3:**
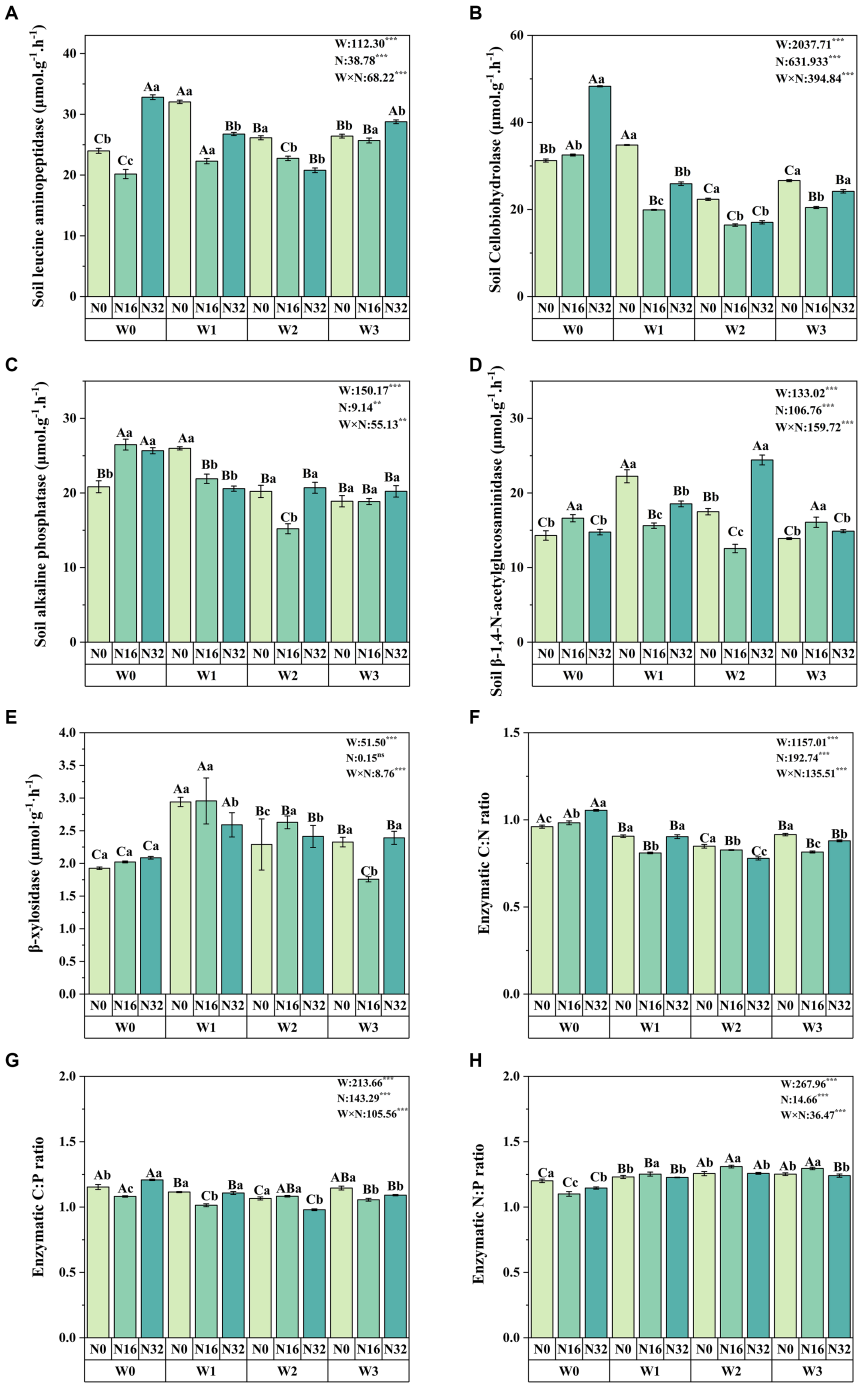
Impact of warming and nitrogen deposition on soil enzyme activity. **(A–E)** Extracellular enzyme activities related to carbon, nitrogen, and phosphorus (soil leucine aminopeptidase, soil cellobiohydrolase, soil alkaline phosphatase, soil β-1,4-N-acetylglucosaminidase, and β-xylosidase). **(F–H)** Soil enzyme stoichiometry (C: N, C:P, and N:P). Values represent the mean ± standard error of the mean for replicated plots (*n* = 4). Duncan’s test indicates significant differences, where different lowercase letters denote significant differences at the same warming level with different nitrogen levels, and different uppercase letters denote significant differences at the same nitrogen level with different warming treatments (*p* < 0.05). W: effect of warming treatment; N: effect of nitrogen treatment; W*N: interaction effect of warming and nitrogen treatments. Asterisks indicate significant differences at different levels (***p < 0.001).

Warming significantly affects soil leucine aminopeptidase, soil cellobiohydrolase, soil alkaline phosphatase, soil β-1,4-N-acetylglucosaminidase, and soil β-xylosidase, while nitrogen deposition also significantly impacts these enzyme levels (Figure 3). Compared to the control group (W0), soil leucine aminopeptidase increased by 36.81, 7.10, and 8.97% in the W1, W2, and W3 treatments, respectively. Soil cellobiohydrolase increased by 11.42% in the W1 treatment, while soil alkaline phosphatase increased by 24.73% in the W1 treatment. Soil β-1,4-N-acetylglucosaminidase increased by 55.57, 22.34, and 32.13% in the W1, W2, and W3 treatments, respectively, and β-xylosidase levels increased by 52.68, 18.90, and 20.81% in the W1, W2, and W3 treatments, respectively. Similarly, compared to the control group (N0), soil leucine aminopeptidase increased by 10.13% in the N32 treatment, while soil cellobiohydrolase increased by 4.09 and 54.62% in the N16 and N32 treatments, respectively. Soil alkaline phosphatase increased by 27.10 and 23.18% in the N16 and N32 treatments, respectively, and soil β-1,4-N-acetylglucosaminidase increased by 16.20 and 3.20% in the N16 and N32 treatments, respectively. Additionally, the interaction between warming and nitrogen deposition significantly influences soil enzyme activity, with soil leucine aminopeptidase reaching its maximum in the W1N0 treatment, while soil cellobiohydrolase and soil alkaline phosphatase reach their peak levels in the W0N32 treatment. Soil β-1,4-N-acetylglucosaminidase reaches its maximum in the W2N32 treatment, and β-xylosidase levels peak in the W1N16 treatment.

Both warming and nitrogen deposition significantly influence soil enzyme C: N, C:P, and N:P ratios. Compared to the control group (W0), soil enzyme C: N ratios decreased significantly by 5.69, 11.69, and 4.73% in the W1, W2, and W3 treatments, respectively, while soil enzyme N:P ratios increased significantly by 2.47, 4.63, and 4.26%. Soil enzyme C:P ratios also decreased significantly by 3.37 and 7.60% in the W1 and W2 treatments, respectively. Similarly, compared to the control group (N0), soil enzyme C: N ratios increased significantly by 2.30 and 9.77% in the N16 and N32 treatments, respectively, while soil enzyme N:P ratios decreased significantly by 8.36 and 4.59%. Additionally, the interaction between warming and nitrogen deposition significantly influences soil enzyme carbon, nitrogen, and phosphorus stoichiometry. Soil enzyme C: N and C:P ratios reach their maximum in the W0N32 treatment, while the soil enzyme N:P ratio reaches its peak in the W2N16 treatment (Figures 3F,G,H).

### Soil microbial diversity and composition

To elucidate the differences in soil microbial diversity and community, we investigated bacterial and fungal diversity and community composition across various levels of warming and nitrogen deposition. Under warming treatment, both the bacterial Chao1 index and Shannon index exhibited significant increases (*p* < 0.05) (Supplementary Figures S1A,B). Specifically, there were notable increases of 2.65 and 0.56% in the W1 treatment, 12.71 and 4.44% in the W2 treatment, and 26.88 and 5.37% in the W3 treatment, respectively. In the N32 treatment, the bacterial Chao1 index and Shannon index increased significantly by 2.30 and 1.25%, respectively. Moreover, the interaction between warming and nitrogen deposition significantly affected the soil bacterial Chao1 index. However, there were no significant changes in the fungal Chao1 index and Shannon index with warming and nitrogen addition (Supplementary Figures S1C,D). ANOSIM results with R > 0 indicate that inter-group differences surpass intra-group differences, with *p* < 0.05 denoting statistical significance. This implies that both warming and nitrogen deposition exert significant influence on soil microbial community structure (Supplementary Figures S2).

**Figure 4:**
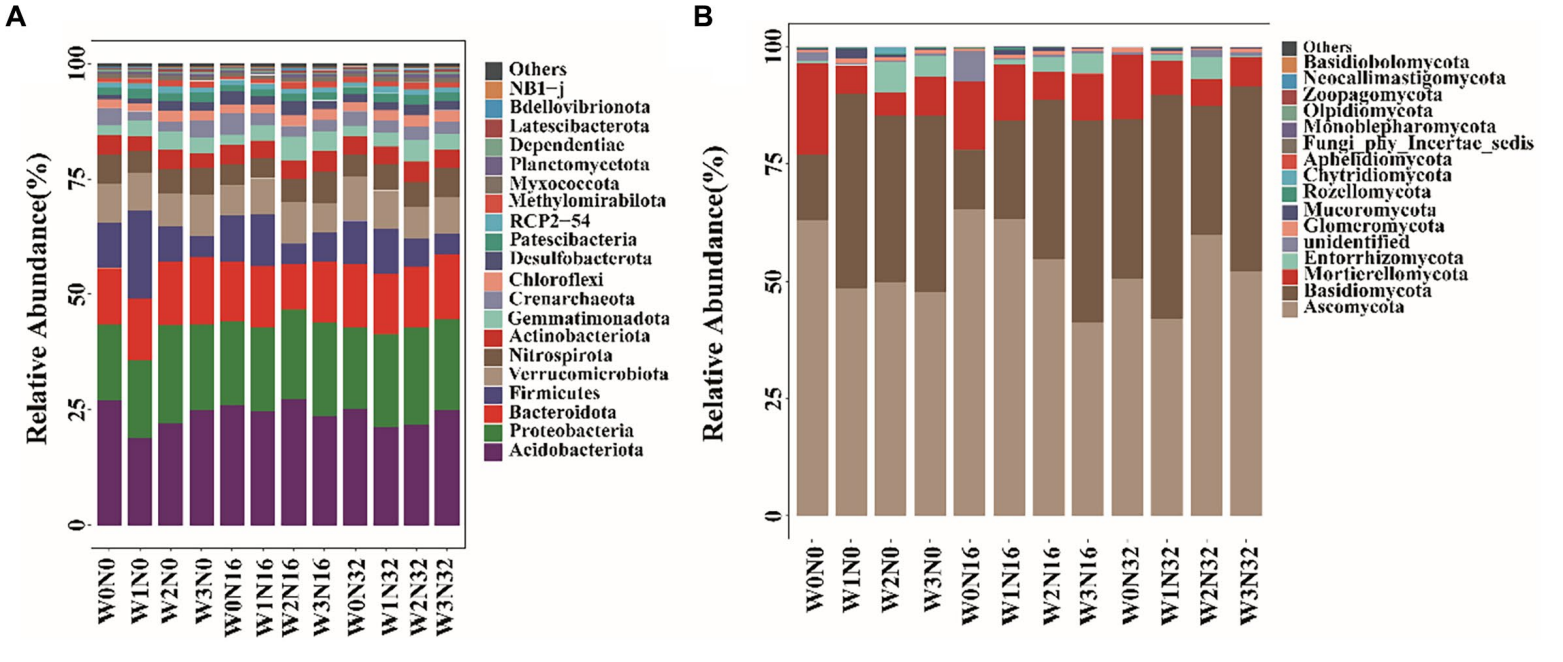
The composition of soil microbial community structure on phylum level under different treatments. **(A)** The situation of soil bacteria. **(B)** The situation of soil fungal.

Differences in bacterial community composition are evident under warming and nitrogen deposition (Figure 4A). The dominant phyla of soil bacteria include *Acidobacteriota*, *Proteobacteria*, and *Bacteroidota*. With warming, the relative abundance of *Acidobacteriota* declined by 8.40–30.10%, whereas Proteobacteria increased from 16.42 to 21.12%, and *Bacteroidota* increased by 10.44–20.04%. Under nitrogen deposition, the relative abundance of *Acidobacteriota* decreased by 4.68–7.37%, while Proteobacteria increased from 16.42 to 18.30%, and *Bacteroidota* increased by 7.09–13.40%. The dominant phyla of soil fungi were *Ascomycota*, *Basidiomycota*, and *Mortierellomycota* (Figure 4B). With warming, the relative abundance of Ascomycota decreased by 21.14–24.34%, while *Basidiomycota* increased from 13.91 to 41.48%. The relative abundance of *Mortierellomycota* decreased by 57.46–74.09%. Under nitrogen addition, the relative abundance of *Ascomycota* initially increased followed by a decrease, Basidiomycota initially decreased followed by an increase, while the relative abundance of *Mortierellomycota* gradually decreased.

### Linkages between soil microbial community, enzyme activity, and carbon fractions

**Figure 5:**
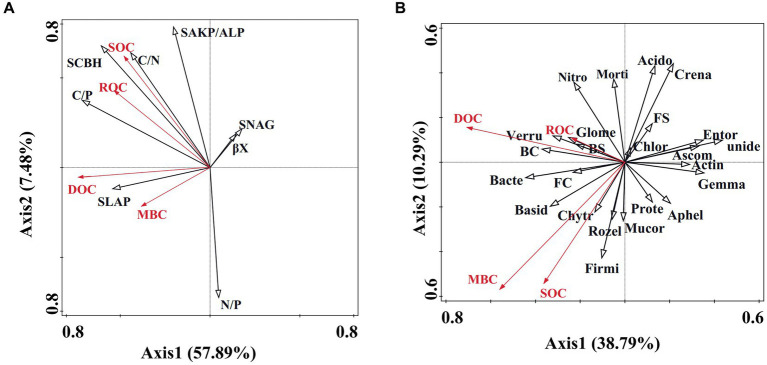
Redundancy analysis of soil microbial community and enzyme activity regarding organic carbon dynamics. **(A)** Redundancy analysis illustrating the correlation between enzyme activity and organic carbon dynamics. SOC, ROC, DOC, and MBC denote soil organic carbon, readily oxidizable carbon, dissolved organic carbon, and microbial biomass carbon, respectively. S-LAP, SCBH, S-AKP/ALP, S-NAG, and cc represent soil leucine aminopeptidase, soil cellobiohydrolase, soil alkaline phosphatase, soil β-1,4-N-acetylglucosaminidase, and β-xylosidase, respectively. **(B)** Redundancy analysis demonstrating the association between soil microbial community and organic carbon dynamics. Unide, Gemma, Crena, Nitros, BC, Chlor, Verru, Ascom, Bacter, Chytr, FC, FS, Entor, Morti, Rozel, Aphel, Acido, Mucor, Firmi, Prote, Actin, Glome, BS, Basid represent unidentified, Gemmatimonadota, Crenarchaeota, Nitrospirota, Bacteria chao, Chloroflexi, Verrucomicrobiota, Ascomycota, Bacteroidota, Chytridiomycota, Fungus chao, Fungus Shannon, Entorrhizomycota, Mortierellomycota, Rozellomycota, Aphelidiomycota, Acidobacteriota, Mucoromycota, Firmicutes, Proteobacteria, Actinobacteria, Glomeromycota, Bacteria Shannon, Basidiomycota.

Redundancy analysis (RDA) results reveal that 57.89% of soil carbon fractions can be elucidated by soil enzyme activity (Figure 5A). Notably, factors such as C/P, S-LAP, S-AKP/ALP, βX, S-NAG, and N/P significantly influence soil organic carbon (Table 2). Furthermore, soil microbial community diversity and structure account for 38.79% of soil organic carbon dynamics (Figure 5B). Within the top 10 microbial taxa at the phylum level, *Gemmatimonadota*, *unidentified*, *Crenarchaeota*, and *Nitrospirota* emerge as pivotal microbial groups (Table 2).

**Table 2:** Monte Carlo permutation test results for redundancy analysis.

Factor	Explains %	*p*	Factor	Explains %	*p*
C/P	31.7	0.002	N/P	7.9	0.002
S-LAP	17.5	0.002	Gemmatimonadota	8.6	0.048
S-AKP/ALP	6.1	0.012	Unidentified	7.8	0.048
βX	3.6	0.021	Crenarchaeota	5.2	0.049
S-NAG	4.0	0.016	Nitrospirota	4.9	0.049

**Figure 6:**
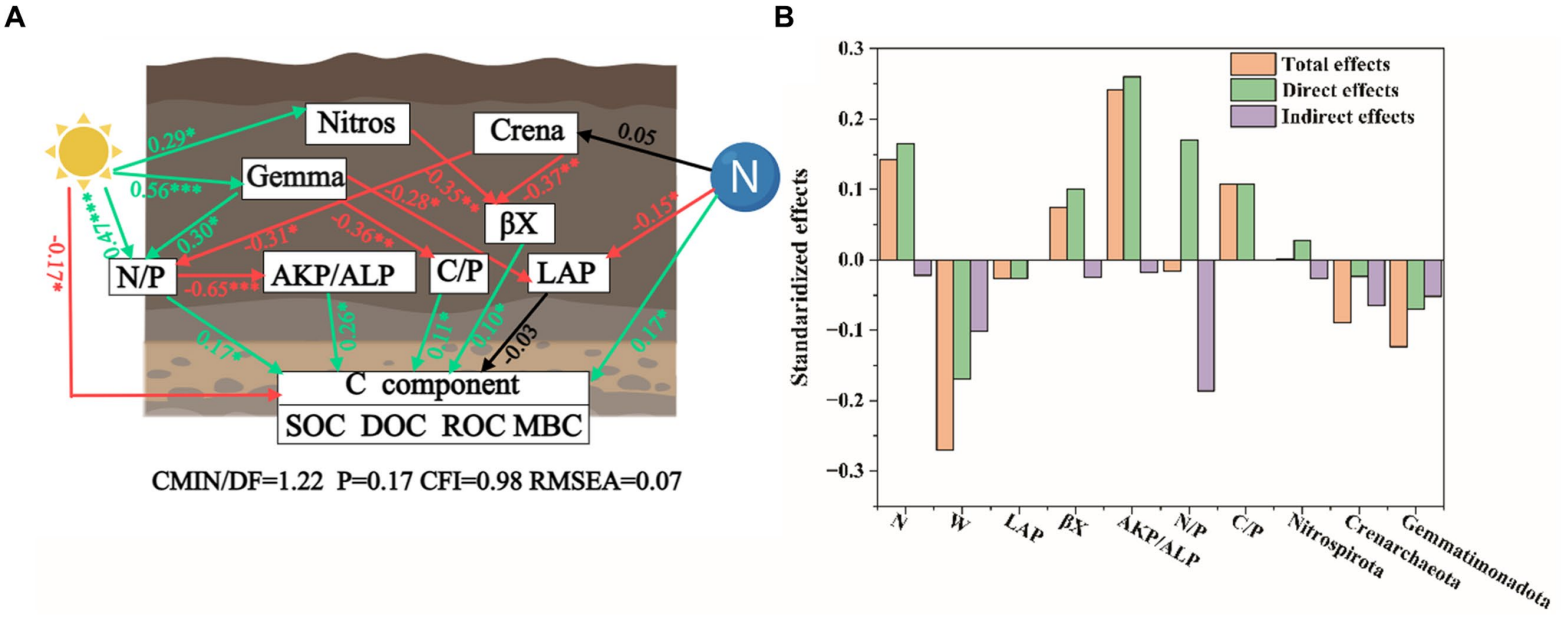
Structural equation model analysis of soil enzyme activity and microbial community on organic carbon under warming and nitrogen deposition. **(A)** Schematic representation of the structural model (Created with MedPeer.com). Green, red, and black colors indicate positive, negative, and no effects, respectively. Asterisks denote significance at probability levels of *p* < 0.01 and *p* < 0.001 (represented as ** and ***). **(B)** Direct and indirect effects of different factors on soil organic carbon fractions.

The results of the structural equation model indicate that changes in soil carbon fractions are directly and indirectly influenced by nitrogen deposition, warming, soil enzyme activity, and soil microbial composition (Figure 6A). Climate warming has a significant positive effect on *Gemmatimonadota*, *Nitrospirota*, and N/P ratio, while it negatively impacts soil carbon fractions. Conversely, nitrogen deposition negatively affects LAP but positively influences soil carbon fractions. In summary, climate warming and nitrogen deposition alter soil microbial composition (such as *Gemmatimonadota*, and *Nitrospirota*), thereby affecting soil microbial enzyme activity (including S-AKP/ALP, βX) and enzyme stoichiometry (N/P ratio, C/P ratio), ultimately leading to changes in soil carbon fractions. The main factors influencing soil carbon fractions under climate warming and nitrogen deposition include S-AKP/ALP, enzyme N/P ratio, nitrogen deposition, enzyme C/P ratio, and βX. The direct effects of temperature increase and nitrogen deposition on soil organic carbon are −0.17 and 0.17, respectively (Figure 6B).

## Discussion

### Impact of warming and nitrogen deposition on soil organic carbon fractions

This study illustrates that nitrogen deposition fosters an increase in soil organic carbon, consistent with findings in the Qinghai-Tibetan Plateau (Xiao et al., 2020) and the Yellow River Delta region (Qu et al., 2020). Nitrogen addition stimulates plant growth and microbial decomposition in nitrogen-limited environments, thereby influencing soil microbial communities and plant decomposition patterns, ultimately affecting soil organic carbon retention (Frey et al., 2013). Temperature is a critical limiting factor in high-altitude ecosystems (Duan et al., 2019). Our study found that warming increases soil organic carbon. Other studies have shown that changes in microbial community structure induced by warming can promote carbon release (Chen et al., 2021). This is mainly due to plant inputs, such as rhizosphere exudates and litter, being significant pathways for soil organic carbon formation. Research suggests that enhanced nitrogen availability can amplify warming’s effects on microbial-mediated litter and organic matter decomposition (Chen et al., 2017b). In our study, soil organic carbon reached its maximum in the W0N32 treatment, indicating that the response of soil organic carbon to nitrogen deposition and warming is not simply additive. A comprehensive assessment of various climatic factors is crucial for understanding changes in soil carbon storage.

Soil microbial biomass carbon serves as an indicator of the microbial biomass involved in decomposition processes and is considered a reliable indicator of soil nutrient cycling potential (Fan et al., 2021). This study demonstrates that elevated temperature and nitrogen deposition significantly influence soil microbial biomass carbon, reaching its peak in the W3N32 treatment. This suggests that warming and nitrogen deposition increase the availability of nutrients in the soil, creating a favorable environment for microbial survival and promoting microbial growth and metabolism, thereby enhancing soil microbial biomass carbon (Chen et al., 2014). Soil-dissolved organic carbon, the most readily utilizable portion of carbon substrates for microbes, is crucial for microbial metabolic maintenance (Fungo et al., 2017). Studies have shown that warming can increase soil dissolved organic carbon by boosting the production of dissolved organic compounds by plants or microbial communities (Bai et al., 2020). Conversely, excessive nitrogen addition can reduce aboveground biomass and microbial activity, impairing microbial decomposition of litter, and leading to a decrease in soil dissolved organic carbon (Zhong et al., 2015). In our study, we found that soil dissolved organic carbon reached its maximum in the W0N32 treatment. This is primarily because nitrogen addition increases plant biomass, with plant residues and secretions being the main sources of soil dissolved organic carbon. Additionally, nitrogen addition promotes plants to provide carbon sources for soil microbes, enhancing microbial activity in the soil, thereby accelerating the decomposition of plant residues, releasing more nutrients, improving soil structure, and promoting soil dissolved organic carbon (Yang et al., 2016). The migration and transformation of soil readily oxidizable organic carbon content also play a crucial role in carbon cycling in grassland ecosystems (Liu F. et al., 2018; Liu H. Y. et al., 2018). Our study found that soil readily oxidizable organic carbon content reached its maximum in the W0N32 treatment, and soil readily oxidizable organic carbon content is the main component of soil organic carbon. A higher ROC/SOC ratio indicates stronger soil organic carbon activity, while a lower ratio indicates more stable soil (Han et al., 2022). Since soil readily oxidizable organic carbon content is mostly composed of recently fallen plant residues, nitrogen addition promotes the decomposition of surface litter by soil microbes, accelerating soil organic carbon turnover rates (Liu F. et al., 2018; Liu H. Y. et al., 2018).

### Impact of warming and nitrogen deposition on soil enzyme activity and stoichiometry

Soil enzymes play a crucial role in regulating key processes such as microbial decomposition of organic matter and soil nutrient cycling (Liu et al., 2021). Previous studies have consistently demonstrated the positive effects of nitrogen addition on C-acquiring, especially β-glucosidase (BG), and P-acquiring, particularly alkaline phosphatase (AP), and hydrolases (Xiao et al., 2018). Our study also observed significant increases in β-xylosidase activity and soil AP in response to nitrogen addition, suggesting that nitrogen stimulates microbial demand for carbon and phosphorus (Yang et al., 2022). Furthermore, nitrogen addition enhances the activity of nitrogen-acquiring enzymes, such as soil leucine aminopeptidase and soil N-acetylglucosaminidase. However, it has been documented that nitrogen addition significantly reduces the activity of soil microbial enzymes involved in carbon, nitrogen, and phosphorus acquisition, thereby impeding the transformation of chemical elements and the decomposition and release of nutrients in the soil (Li et al., 2021). Consequently, the response of soil enzyme activity to nitrogen deposition is multifaceted and should take into account soil nutrient status, microbial conditions, nitrogen addition forms, plant community composition, and grassland type (Margalef et al., 2021).

Elevated temperature also impacts soil enzyme activity in various ways. Firstly, it promotes the binding of soil enzymes to substrates, thereby altering their activity. Secondly, temperature influences the mineralization of soil organic matter, accelerates litter decomposition, and induces changes in microbial communities, indirectly affecting enzyme activity (Morrison et al., 2019). Our study observed significant effects of warming on soil leucine aminopeptidase, soil cellobiohydrolase, soil alkaline phosphatase, soil β-1,4-N-acetylglucosaminidase, and β-xylosidase. Prior research has also noted increased activities of β-glucosidase (βG) and cellobiohydrolase (CBH) under elevated temperatures (Jiang et al., 2018). This can be attributed to the facilitation of microbial mobility, nutrient movement, and enzyme-substrate binding in soil solutions under higher temperatures, provided there is sufficient water availability (Alvarez et al., 2018). Studies have shown that the interaction between temperature and nitrogen addition significantly enhances the activity of soil acid phosphatase (aG), βG, CBH, and alkaline phosphatase (ACP) (Huang et al., 2022). In our investigation, β-xylosidase and soil alkaline phosphatase peaked in the W0N32 treatment, while soil N-acetylglucosaminidase reached its maximum in the W2N32 treatment. Additionally, minimal or insignificant changes in soil phosphatase, urease, and β-glucosidase activity have been reported due to climate warming (Pold et al., 2017; Souza et al., 2017). Furthermore, elevated temperatures exacerbate transpiration, leading to reduced soil moisture content, which hampers the effectiveness of soil enzyme substrates (Liu W. X. et al., 2009). This indicates that different enzymes have distinct optimal temperature ranges, with excessively high temperatures causing denaturation and excessively low temperatures reducing activity.

Ecological enzyme stoichiometry, derived from soil carbon, nitrogen, and phosphorus extracellular enzyme activities, serves as a valuable tool for elucidating microbial resource constraints across various ecosystems (Xu et al., 2022). Our findings reveal that nitrogen deposition leads to a significant increase in soil enzyme C: N ratios, coupled with a notable decrease in soil enzyme N:P ratios. This trend suggests that warming and nitrogen deposition exacerbate microbial phosphorus limitation (Ma et al., 2020). The intensified microbial phosphorus limitation is linked to nitrogen addition fostering plant growth, consequently heightening plant demand for and uptake of phosphorus, thereby intensifying nutrient competition between plants and microbes (Liu et al., 2022). Concurrently, increased soil temperature modifies soil nutrient turnover and microbial activity, consequently influencing soil extracellular enzyme stoichiometry. Specifically, our results indicate that under warming conditions, soil enzyme C: N and C:P ratios exhibit significant decreases, while soil enzyme N:P ratios register significant increases. This pattern arises from elevated temperatures accelerating cell membrane maintenance and lipid turnover, thereby augmenting microbial demand for nitrogen and phosphorus (Fang et al., 2016). Moreover, our study demonstrates that soil enzyme C: N and C:P ratios peak in the W0N32 treatment, while soil enzyme N:P ratios reach their maximum in the W2N16 treatment. This phenomenon may stem from soil microbial communities adjusting their extracellular enzyme production to achieve chemical stoichiometric balance in response to changes in nutrient availability. To maintain elemental stability, microbes can flexibly modulate the production of their extracellular enzymes by maximizing the mobilization of substrates abundant in limiting elements (Mooshammer et al., 2014).

### Impact of warming and nitrogen deposition on soil microbial composition and structure

Microorganisms play a pivotal role in ecosystem functions, including soil nutrient cycling and organic matter decomposition (Luo et al., 2020). This study illustrates the influence of warming and nitrogen deposition on soil bacterial community diversity, as measured by Shannon and Chao1 indices, while soil fungal diversity remains largely unchanged. These findings suggest that bacterial communities undergo sustained alterations under climate change, whereas soil fungi exhibit relative stability. This phenomenon can be attributed to two main factors: (1) Direct modification of soil microbial community composition by climate warming, driven by the diverse optimal growth temperatures of different microbial species (Fukasawa, 2018). (2) Indirect impacts of warming and nitrogen deposition on plant communities and soil nutrient status, thereby influencing soil microbial community diversity (Liu F. et al., 2018; Liu H. Y. et al., 2018). Bacteria and fungi demonstrate variations in metabolic adaptability, resulting in diverse impacts on soil microbial activity (Begum et al., 2019).

Research indicates that nitrogen addition can lead to a decrease in the abundance of specific bacterial phyla, including *Actinobacteria*, *Proteobacteria*, and *Acidobacteria* (Freedman et al., 2016). According to the oligotrophic symbiotic nutrition theory, symbiotic taxa exhibit high nutrient requirements and growth rates, whereas oligotrophic taxa can survive in environments with low organic carbon availability (Xie et al., 2019). *Acidobacteria* are recognized as oligotrophic microbes, while Proteobacteria are categorized as copiotroph microbes (Fierer et al., 2007). In this investigation, both nitrogen addition and temperature elevation resulted in a decreased abundance of *Acidobacteriota* and an increased abundance of Proteobacteria. Consequently, the ratios of oligotrophic to copiotrophic (o: c) and acidobacteria to proteobacteria (a: P) remained unchanged under warming and nitrogen treatments. This study reveals that both warming and nitrogen deposition significantly diminished the relative abundance of *Mortierellomycota* but had no discernible impact on soil fungal microbiota. Previous research has similarly indicated that warming did not affect soil fungal diversity but did reduce the complexity of soil fungal communities (Yu et al., 2024). Nitrogen enrichment can also influence soil microbial community structure, although its effects on diversity and abundance are minimal (Wang et al., 2023). These findings suggest that the response of soil microbial communities to the altered soil environment resulting from climate warming and nitrogen deposition is intricate (Mitchell et al., 2015).

### Impact of soil microbes and enzyme activity on carbon composition under climate warming and nitrogen deposition

Soil microbes represent a significant portion of global biodiversity and play essential roles in carbon sequestration, organic matter decomposition, and nutrient cycling (Luo et al., 2020). Their extracellular enzymes are pivotal in regulating the rate of organic matter decomposition (Yang et al., 2022). Studies have shown that elevated soil temperatures can enhance the activity of soil N-acetylglucosaminidase and alkaline phosphatase by altering the microbial community composition, thus influencing soil carbon decomposition and transformation (Zhou et al., 2013). The findings of this study reveal that climate warming and nitrogen deposition reshape soil microbial composition, notably affecting soil microbial enzyme activity, such as AKP/ALP and βX, and enzyme stoichiometry, including the N/P ratio and C/P ratio, consequently altering soil carbon composition. This is attributed to the selection of different soil microbial communities under climate warming and nitrogen deposition, which further reshapes soil ecosystem processes and functions (Li et al., 2018). Enzymes related to carbon, nitrogen, and phosphorus are primarily produced by microbes, and variations in microbial quantity and types can lead to changes in enzyme activity levels. Elevated extracellular enzyme activity levels can degrade both unstable and recalcitrant carbon substances, thereby influencing the biotransformation processes of organic carbon (Sardans et al., 2017).

This study reveals that soil organic carbon composition significantly decreases with climate warming, attributed to changes in microbial community composition or substrate utilization efficiency, accelerating microbially mediated organic matter degradation and reducing soil organic carbon content (Chen et al., 2017a). Additionally, soil organic carbon composition is directly influenced by nitrogen. This is due to two main reasons: firstly, nitrogen can enhance soil organic carbon accumulation by promoting plant growth, increasing litterfall, and inhibiting microbial degradation of soil organic matter (Xia et al., 2017). Secondly, the nitrogen addition threshold for grasslands on the Qinghai-Tibet Plateau is 272 kg N ha^−1^ year^−1^ (He et al., 2024). Studies indicate that nitrogen inputs below this critical level can suppress soil respiration by inhibiting soil microbial growth, reducing root biomass, and litter decomposition, thus fostering soil carbon sequestration (Xiao et al., 2021). Therefore, nitrogen inputs ranging from 16 to 32 kg N ha^−1^ year^−1^ may promote carbon sequestration in alpine meadows on the Qinghai-Tibet Plateau.

## Conclusion

Both climate warming and nitrogen deposition significantly increased soil organic carbon components, including organic carbon content, readily oxidizable carbon, dissolved organic carbon, and microbial biomass carbon. Moreover, soil enzyme activity, such as Soil Cellobiohydrolase, β-1,4-N-acetylglucosaminidase, leucine aminopeptidase, and alkaline phosphatase, was enhanced under these conditions. Similarly, soil bacterial diversity, as indicated by the Chao1 index and Shannon index, also showed significant increments. Additionally, climate warming led to a decrease in the soil enzyme C: N ratio and C:P ratio, while increasing the soil enzyme N:P ratio. Conversely, nitrogen deposition resulted in a significant increase in the soil enzyme C: N ratio and a decrease in the soil enzyme N:P ratio. The soil organic carbon components were directly influenced by the negative impact of climate warming and the positive impact of nitrogen deposition. Furthermore, climate warming and nitrogen deposition altered soil bacterial, particularly *Gemmatimonadota*, and *Nitrospirota*, leading to a positive impact on soil enzyme activity, particularly soil alkaline phosphatase and β-xylosidase, and enzyme stoichiometry, including C:P and N:P ratios.

## Data availability statement

The datasets presented in this study can be found in online repositories. The raw sequence reads can be found at: https://www.ncbi.nlm.nih.gov/bioproject/PRJNA1096318.

## Author contributions

XX: Data curation, Formal analysis, Investigation, Software, Writing – original draft. DK: Funding acquisition, Writing – review & editing. LW: Formal analysis, Investigation, Writing – review & editing. FT: Formal analysis, Investigation, Writing – review & editing. LF: Formal analysis, Investigation, Writing – review & editing. WX: Formal analysis, Investigation, Writing – review & editing.
