# Supplementary material for: Indirect influence of soil enzymes and their stoichiometry on soil organic carbon response to warming and nitrogen deposition in the Tibetan Plateau alpine meadow
Source: Front Microbiol. 2024 Apr 17;15:1381891. doi: 10.3389/fmicb.2024.1381891 (PMC11061507; doi:10.3389/fmicb.2024.1381891)
Supplement: Supplementary file 1 [file Presentation_1.pdf]

Appendix A Supplementary Data

Indirect Influence of Soil Enzymes and Their Stoichiometry on Soil Organic Carbon Response to Warming and Nitrogen Deposition in the Tibetan Plateau Alpine Meadow

Xiang Xuemei, De Kejia* , Lin Weishan, FENG Tingxu, Li Fei, WEI Xijie
College of Animal Husbandry and Veterinary Science, Qinghai University, Qinghai Province, Xining 810010, China

* Corresponding author. E-mail address: dekejia1002@163.com

TABLE S1 Effects of Warming, Nitrogen Deposition, and Their Interaction on the Ratios of Soil Carbon Fractions

Treatment		DOC/SOC(%)	MBC/SOC(%)	ROC/SOC(%)
	N0	0.35±0.01Ba	5.60±0.26Ba	22.6±0.26Ba
W0	N16	0.21±0.02Bb	5.35±0.1C4b	21.6±0.40Bb
	N32	0.35±0.04Aa	5.20±0.90Bb	22.6±0.53Aa
	N0	0.28±0.07Ca	5.73±0.12Bb	19.5±0.51Ca
W1	N16	0.25±0.02Bb	6.16±0.44Ba	18.7±0.38Cb
	N32	0.23±0.06Bb	6.04±0.12Ab	18.0±0.23Cb
	N0	0.31±0.05Ba	7.11±0.11Aa	21.6±0.30Bb
W2	N16	0.25±0.07Bb	6.53±0.1A8b	26.5±0.64Aa
	N32	0.27±0.03Bb	5.68±0.50Bc	20.2±0.33Bb
	N0	0.41±0.04Aa	6.94±0.02Aa	23.8±0.14Aa
W3	N16	0.33±0.05Ab	6.78±0.01Aab	23.0±0.51Ba
	N32	0.35±0.05Ab	6.31±0.01Ab	21.4±0.98Ab
W		603.08^{***}	120.02^{***}	95.22^{***}
N		579.19^{***}	38.47^{***}	32.65^{***}
W*N		116.30^{***}	16.31^{***}	26.39^{***}

Note: Lowercase letters indicate significant differences among different nitrogen levels under the same warming treatment, while uppercase letters indicate significant differences among different warming treatments under the same nitrogen level (n=4). W: Effect of warming treatment; N: Effect of nitrogen treatment; W*N: Interaction effect of warming and nitrogen treatments. *P < 0.05; ** P < 0.01; ***P < 0.001; ns: Not significant.

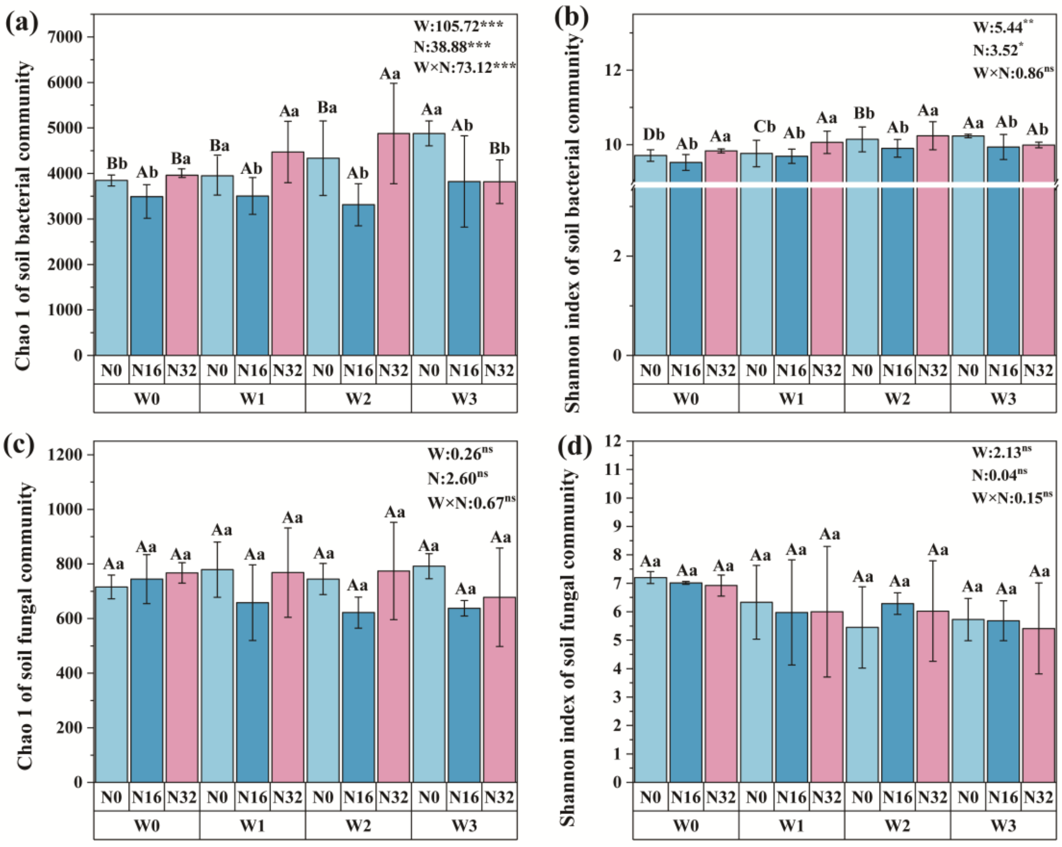

FIGURE S1 Soil Microbial Community Alpha Diversity under Warming and Nitrogen Deposition. (a) and (b) depict the Chao1 and Shannon indices of soil bacteria, respectively, while (c) and (d) represent the Chao1 and Shannon indices of soil fungi, respectively.

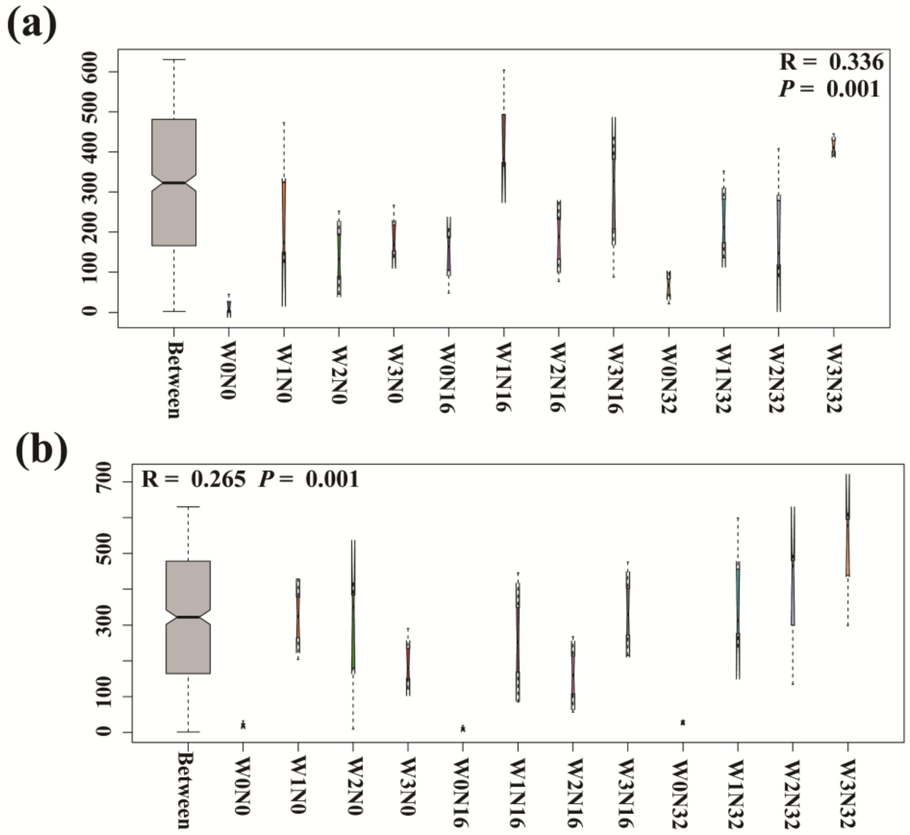

FIGURE S2 ANOSIM Results Based on Bray-Curtis Distance. (a) and (b) depict the ANOSIM results for soil bacteria and fungi, respectively.
